# Finite Element Calculation with Experimental Verification for a Free-Flooded Transducer Based on Fluid Cavity Structure

**DOI:** 10.3390/s18093128

**Published:** 2018-09-17

**Authors:** Zhengyao He, Geng Chen, Yun Wang, Kan Zhang, Wenqiang Tian

**Affiliations:** School of Marine Science and Technology, Northwestern Polytechnical University, Xi’an 710072, China; chengeng@mail.nwpu.edu.cn (G.C.); wygdbzwrh@mail.nwpu.edu.cn (Y.W.); zhangkannpu@mail.nwpu.edu.cn (K.Z.); tianwq@mail.nwpu.edu.cn (W.T.)

**Keywords:** free-flooded transducer, fluid cavity structure, finite element model, acoustic radiation

## Abstract

A free-flooded transducer that couples the vibration of a longitudinal vibration transducer and the fluid cavity of an aluminum ring was investigated. Given the transducer is based on a fluid cavity structure and has no air cavity, it can resist high hydrostatic pressure when working underwater, which is suitable for application in the deep sea. At first, the structure and working principle of the transducer were introduced. Then, the axisymmetric finite element model of the transducer was established; and the transmitting voltage response, admittance, and radiation directivity of the transducer were simulated using the finite element method. According to the size of the finite element model, a prototype of the transducer was designed and fabricated, and the electro-acoustic performance of the prototype was measured in an anechoic water tank. The experimental results were consistent with the simulation results and showed a good performance of the transducer. Finally, the improvement of the radiation directivity of the transducer by the optimal design of the free-flooded aluminum ring was obtained using the finite element method and verified by experiments.

## 1. Introduction

Since ocean exploration is developing towards the deep sea, underwater acoustic transducers require the performance of low frequency and high power in deepwater. The free-flooded structural transducers have good performance in deepwater, with low resonant frequency and high transmitting response [1]. Piezoelectric ceramic rings and tubes are often applied as deepwater transducers, because their characteristics are essentially independent of depth [2,3,4]. Free-flooded ring transducers composed of piezoelectric ceramic rings are studied [2,3]. A deepwater tube transducer, which transmits signals by mechanically tuning a resonator tube is researched [4]. The Helmholtz resonant transducers are studied for their high-power deepwater use [5,6,7]. This paper investigated a transducer with a free-flooded structure that couples the vibration of a longitudinal vibration transducer and the fluid cavity of an aluminum ring, which is suitable for application in the deep sea. It can be used in deepwater sonar detection, deepwater acoustic communication, and sound propagation characteristics research in deep sea, etc.

The finite element method is a modeling and analysis method of transducers commonly used in recent years. It uses a series of discrete units to represent and model the transducer structure and fluid domain, as such its outstanding advantage is that it can adapt to complex conditions, such as irregular boundary shapes, inhomogeneous materials, and anisotropy; and can perform modeling and calculation on transducers of complex structure [8,9,10]. Using the finite element method to model the transducer we can easily calculate the resonant frequency, observe the displacement distribution of the transducer at resonance, and obtain the admittance curve, transmitting voltage response curves, and directivity pattern of the transducer [10]. It can also be used to optimize the structure of transducers. The acoustic radiation characteristics of free-flooded ring transducers are calculated using the finite element method [2]. The Class IV flextensional transducer is modeled and calculated using the finite element method in ANSYS [10]. The Class III barrel-stave flextensional transducer coupling the fundamental flexural and longitudinal modes of vibration is calculated using the finite element analysis techniques [11]. In this paper, the finite element method was used to model and calculate the free-flooded transducer based on fluid cavity structure, where the influence of the free-flooded aluminum ring on radiation directivity is analyzed, the size of the transducer is optimally designed, and the performance is verified by experiments in the anechoic water tank.

## 2. Structure of the Transducer

**Figure 1:**
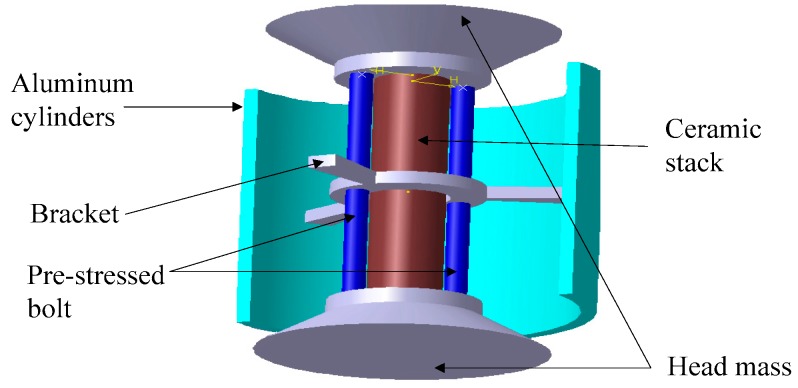
The structure of the transducer.

The structure of the free-flooded transducer based on fluid cavity structure is shown in Figure 1. This kind of transducer is free-flooded with a fluid cavity, which has no air cavity. It can resist high hydrostatic pressure when working underwater, which is suitable for application in the deep sea. The transducer couples the vibration of a longitudinal vibration transducer and the fluid cavity of an aluminum ring. The longitudinal vibration transducer is stacked using 24 pieces of PZT-4 piezoelectric ceramic. The two head mass, which are made of stainless steel material, are an inverted frustum of a cone. The longitudinal vibration transducer in this structure can obtain a high transmitting voltage response. When the parameters of the transducer are optimally designed, the longitudinal resonant frequency is close to the cavity resonant frequency; so the resonance of the fluid cavity of the free-flooded aluminum ring couples with the longitudinal vibration transducer, and a higher transmitting voltage response can be obtained.

The resonant frequency in the fluid cavity of the aluminum ring is given by Equation (1) as shown in Reference [12]:

(1) $$f_{1}=\frac{c}{2\pi r}{\left(1+\frac{\rho_{0}\sqrt{\frac{\mathrm{rH}}{2}}}{\rho t}\right)}^{-1/2}$$

In this formula, r=59 mm is the radius of the aluminum ring, $\rho_{0}=1000\;{\mathrm{kg}/m}^{3}$ is the density of water, ρ = 2700 kg/m³ is the density of aluminum, H=110 mm is the height of the aluminum ring, t=2 mm is the thickness of the aluminum ring, and c=5200 m/s is the speed of sound in aluminum, so that the calculated cavity resonant frequency is $f_{1}=4040\;\mathrm{Hz}$.

The resonant frequency of longitudinal vibration is given by Equation (2) as shown in Reference [12]:

(2) $$f_{2}=\frac{1}{2\pi }\sqrt{\frac{K_{m}}{M_{m}}}$$

In the formula, km is the stiffness of the piezoelectric ceramic stack, and Mm is the dynamic mass. According to the size of the longitudinal vibration transducer, the longitudinal resonant frequency of the transducer is 4156 Hz, which is close to the above cavity resonant frequency.

## 3. Finite Element Modeling and Principle of Operation of the Transducer

**Figure 2:**
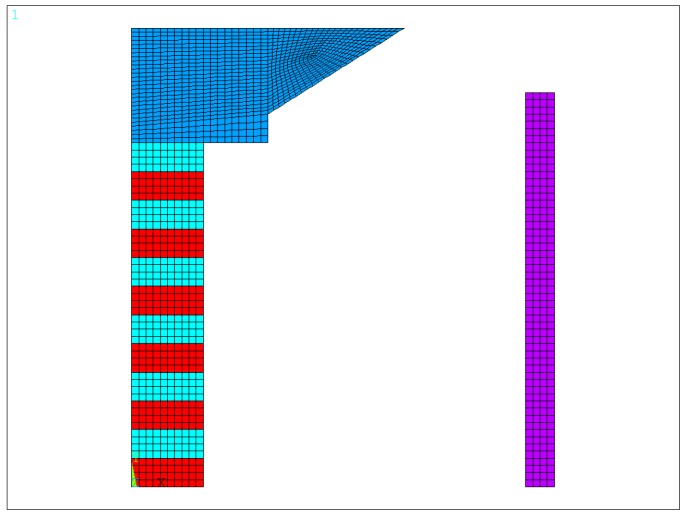
The finite element model of the transducer.

Since the transducer is axisymmetric, and symmetric up and down, the 2-D axisymmetric finite element model can be used to calculate the transmitting characteristics of the transducer. Only the upper half of the transducer needs to be modeled, as shown in Figure 2. The symmetric conditions were applied to improve the simulation efficiency. The piezoelectric ceramic stack consisted of 24 pieces of PZT4 ceramics. The thickness of each ceramic was 4 mm and the radius was 10 mm. The polarization direction was the Y-axis direction, which was the longitudinal direction. The adjacent two ceramics had the opposite polarization direction. The driving voltages were exerted on the piezoelectric ceramics. The head mass of the transducer was made of steel, the maximum radius was 38 mm, and the height was 16 mm. The free-flooded ring was made of aluminum, the inner radius of the ring was 60 mm, the height was 100 mm, and the thickness was 8 mm. In the ANSYS software, the geometry of the transducer was drawn using the above size, and then meshed to obtain the finite element model. In the model, the density, Young’s modulus and Poisson’s ratio of steel and aluminum materials, the density, dielectric constant, piezoelectric constant, and elastic constant of piezoelectric ceramics, and the density and sound velocity of the fluid are needed. In addition, the piezoelectric ceramics were assigned by Plane13 element; the aluminum ring and steel head mass by Plane42; the water area by Fluid29; and the outermost water layer by Fluid129 to simulate the nonreflecting boundary; which were all axisymmetric elements. The radius of the fluid area should satisfy the far-field condition and be selected as large as possible to achieve a high precision of calculation. The condition which should usually be satisfied is:

(3) $$R>\frac{D}{2}+0.2\lambda$$

where R is the distance from the center of the transducer to the outermost boundary of the fluid area, D is the maximum size of the transducer structure, and λ is the sound wavelength.

The acoustic radiation characteristics of the transducer in water can be calculated by harmonic response analysis in ANSYS, based on the finite element model. The transmitting voltage response (TVR) of the transducer is calculated by:

TVR = 20 × log(p(r) × r/p_ref_) (4)

where p(r) is the far-field pressure generated at r meter range by the transducer in water when the driving voltage is 1 V, which can be collected in the finite element model directly, p_ref_ = 1 µPa is the reference pressure.

## 4. Finite Element Analysis and Experimental Verification of the Transducer 

**Figure 3:**
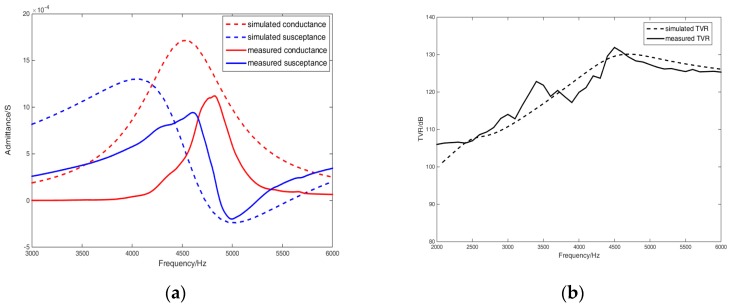
(**a**) The simulated and measured admittance results of the transducer. The solid red and blue line, respectively, represent the measured conductance and susceptance. The dotted red and blue line, respectively, represent the simulated conductance and susceptance. (**b**) The simulated and measured transmitting voltage response of the transducer. The solid and dotted line are the measured and simulated results, respectively.

The finite element model of the free-flooded transducer based on fluid cavity structure is shown in Figure 2. The admittance simulation curve of the transducer is shown by the dotted line in Figure 3a, and the transmitting voltage response curve of the transducer is shown by the dotted line in Figure 3b. The admittance and transmitting voltage response of the transducer were measured in the anechoic water tank, and the measured results are shown by the solid lines in Figure 3a,b, respectively. The resonant frequency of the transducer was 4650 Hz from the admittance simulation results by the finite element method. The maximum transmitting voltage response was 129.5 dB at 4650 Hz by simulation. The resonant frequency of the transducer was 4700 Hz from the experimental admittance results. The maximum transmitting voltage response was 130 dB at 4700 Hz by experiment. The admittance and transmitting voltage response results of the transducer by finite element simulation were consistent with those by experiment.

**Figure 4:**
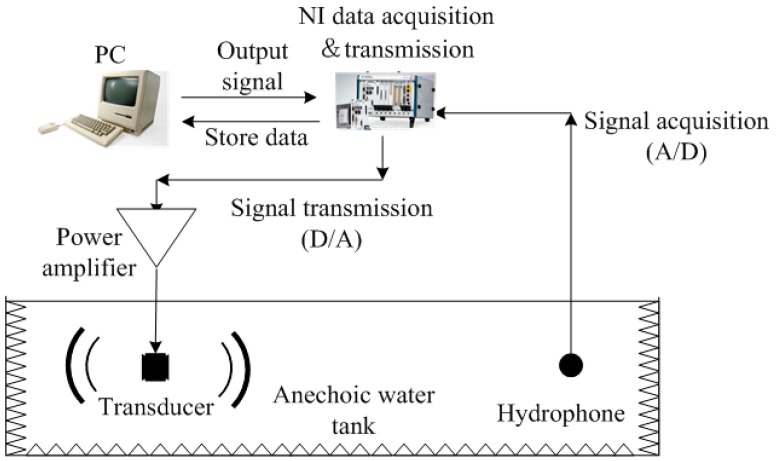
The diagram of the experiment.

The experiments were carried out in an anechoic water tank. The experiment diagram is shown in Figure 4. The computer generated the signals we needed and sent them to the data acquisition and transmission device. Then, the data acquisition and transmission device converted the digital signal to an analog signal, and the signal was outputted to the transducer after power amplifying. The transducer transmitted the signal as an acoustic wave. Then, the hydrophone received the acoustic signal and transformed it into an electrical signal. The data acquisition and transmission device converted the analog signal into a digital signal and stored it in the computer for later data processing. Thus, the transmitting voltage response and radiation directivity could be calculated. The hydrophone we used was BK8104 with a sensitivity of −205 dB, at the frequency range of 10 Hz–10 kHz. The distance between the transducer and hydrophone was 8.3 m, which satisfied the far-field condition.

## 5. Optimization of Radiation Directivity of the Transducer

**Figure 5:**
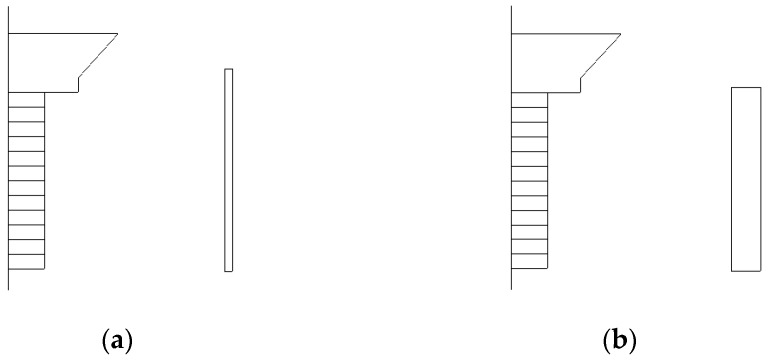
(**a**) The size of the aluminum ring before optimization. (**b**) The size of the aluminum ring after optimization.

**Figure 6:**
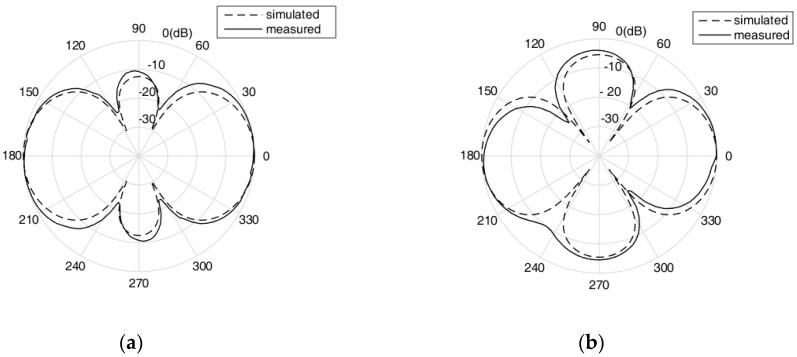
(**a**) The simulated and measured results of the radiation directivity before optimization. (**b**) The simulated and measured results of the radiation directivity after optimization. The solid and dotted lines are the measured and simulated results, respectively.

In the modeling and simulation of the transducer, it was found that the size of the aluminum ring around the outside of the transducer could not only change the transmitting voltage response of the transducer, but also adjust the radiation directivity of the transducer. Through appropriate optimization of the size of the aluminum ring, the radiation directivity of the transducer could be improved. Two kinds of size of the aluminum ring were designed and the performances were compared. Figure 5 shows the size of the aluminum ring before and after optimization. The thickness of the aluminum ring was changed from 2 mm to 8 mm, the radius was changed from 59 mm to 60 mm, and the total height was changed from 110 mm to 100 mm after optimization. Figure 6 shows the simulated and measured results of the radiation directivity of the transducer before and after optimization, at the resonant frequency of 4700 Hz. The 0° and 90° direction were the longitudinal and lateral direction of the transducer, respectively. The radiation directivity results simulated by the finite element method were consistent with the experimental results. The directivity of the transducer was improved after structure optimization, the beam pattern in the 90° direction was broadened and the transmitting response was higher in that direction, which is considered more useful in practical application. It verified the correctness of using the finite element method to simulate radiation directivity and improving the directivity by structure optimization of the transducer.

## 6. Conclusions

In this paper, the finite element method was used to calculate the transmitting voltage response, admittance curve, and radiation directivity of the free-flooded transducer based on fluid cavity structure, which was suitable for application in the deep sea. The influence of the size of the free-flooded aluminum ring on the radiation directivity of the transducer was also analyzed. A prototype of the transducer was designed and fabricated, and experimental measurements were carried out in an anechoic water tank. The theoretical calculation results were consistent with the experimental results. It was verified that the finite element model was correct and feasible for the calculation and analysis of the transducer. The free-flooded transducer based on fluid cavity structure in this paper had good performance, and radiation directivity was improved by the optimal design of the transducer.
